# Case Series of 6 Fetuses With Osteogenesis Imperfecta Type II: A Retrospective Study of Heart Pathology

**DOI:** 10.1177/10935266241272511

**Published:** 2024-08-27

**Authors:** Sara J. E. Verdonk, Silvia Storoni, Lidiia Zhytnik, Dimitra Micha, Joost G. van den Aardweg, Otto Kamp, Elisabeth M. W. Eekhoff, Marianna Bugiani

**Affiliations:** 1 Department of Endocrinology and Metabolism, Amsterdam University Medical Center, Amsterdam, The Netherlands; 2 Rare Bone Disease Center Amsterdam, Amsterdam, The Netherlands; 3 Amsterdam Movement Sciences, Amsterdam, The Netherlands; 4 Department of Human Genetics, Amsterdam University Medical Center, Vrije Universiteit Amsterdam, The Netherlands; 5 Department of Traumatology and Orthopaedics, The University of Tartu, Tartu, Estonia; 6 Amsterdam Reproduction and Development, Amsterdam, The Netherlands; 7 Department of Respiratory Medicine, Amsterdam University Medical Center, Academic Medical Center, Amsterdam, The Netherlands; 8 Department of Cardiology, Amsterdam University Medical Center, Vrije Universiteit, Amsterdam, The Netherlands; 9 Department of Pathology, Amsterdam University Medical Center, Academic Medical Center, Amsterdam, The Netherlands

**Keywords:** osteogenesis imperfecta, collagen type I, heart pathology, OI type II, COL1A1, COL1A2

## Abstract

**Introduction:**

Osteogenesis imperfecta (OI) is a rare genetic disorder characterized by bone fragility. While skeletal manifestations are well documented, few studies have explored the effect of OI on the fetal heart. This retrospective case series investigates cardiac pathology in OI type II fetuses, aiming to address this gap.

**Methods:**

Medical records and autopsy reports of 6 genetically confirmed OI type II cases were examined. Fetuses had pathogenic variants in *COL1A1* or *PPIB*, inducing structural defects in collagen type I. In addition to hematoxylin and eosin and Elastic van Gieson staining, the expression of collagen type I, COL1A1 and COL1A2 chains was examined by immunohistochemistry.

**Results:**

Immunohistochemistry confirmed robust expression of collagen type I throughout the heart. Five fetuses had normal heart weight, while 1 had a low heart weight in the context of generalized growth retardation. None displayed structural heart anomalies.

**Conclusion:**

This study reveals robust collagen type I expression in the hearts of OI type II fetuses without structural anomalies. We hypothesize that collagen type I abnormalities may not be causative factors for heart anomalies during early embryonic development. Instead, their impact may be conceivably related to an increased susceptibility to degenerative changes later in life.

## Introduction

Osteogenesis imperfecta (OI) is a rare genetic disorder characterized by abnormal production of collagen type I, leading to bone fragility and skeletal deformities. Among the various types, OI type II is the most severe form, presenting fractures and severe bone deformities during prenatal development.^1^ OI type II leads to fetal or early postnatal demise. While extensive research has focused on the skeletal manifestations of OI, limited attention has been given to the potential impact of this condition on other organs, including the cardiovascular system, especially during the embryonic stage. Adult individuals with OI have a greater risk of cardiovascular disorders, including heart failure and valvular insufficiency, compared to the reference population.^2^ However, many uncertainties still exist about the underlying pathophysiology, including the developmental versus degenerative nature of heart involvement. Collagen type I is highly expressed in the fetal heart,^3^ including myocardium^4^ and fetal valves.^5^ The scarce literature regarding post-mortem examination of OI type II cases reports OI fetuses with and without cardiovascular anomalies.^6-10^

OI results from genetic alterations leading to the abnormal formation of collagen type I. The collagen type I protein is made up by 2 COL1A1 chains and 1 COL1A2 chain, which are encoded by the *COL1A1* (MIM#120150) and *COL1A2* (MIM#120160) genes, respectively.^11^ The majority of OI cases stem from autosomal dominant variants in either *COL1A1* or *COL1A2.*^12^ The remaining cases are mainly caused by recessive variants in genes involved in post-translational modifications, ensuring the accurate folding of collagen type I.^13,14^ OI type II is associated with pathogenic variants in the *COL1A1*, *COL1A2*, *CRTAP* (MIM#605497), *PPIB* (MIM#123841), *P3H1* (MIM#610339), and *CREB3L1* (MIM#616215) genes. Defects in any of these genes result in severe disruption in the function of the collagen type I protein, in many cases due to altered post-translational modification.^13^

To study the effect of a severe collagen type I abnormalities on the cardiovascular system, we retrospectively investigated the post-mortem features of 6 fetuses with OI type II. Given the scarcity of existing data, our primary focus was to assess the presence and nature of potential cardiovascular anomalies in these cases.

## Materials and Methods

Post-mortem examination reports and formalin-fixed and paraffin-embedded (FFPE) heart tissue sections from 6 fetal OI type II fetuses were retrieved from the Department of Pathology at Amsterdam UMC. In all fetuses, pregnancy was terminated due to the clinical diagnosis of OI type II with ultrasound. Subsequently the diagnosis was confirmed by genetic analysis (Table 1). Consent from the parents was secured in accordance with the guidelines of the Amsterdam Medical Centrum Ethical committee. Pulmonary tissue analyses of these fetuses has been previously described.^15^

**Table 1.** Features of OI Type II Fetuses.

Anonymized code	Gender	Gestational age (week + days)	DNA analysis (HGVS nomenclature)	Heart weight in grams (percentile for gestational age)	Heart macroscopy	Heart microscopy
Fetus 001	M	14 + 5	*COL1A1* c.1543G>C p.(Gly515Arg) (heterozygous) (ref. seq. NM_000088.3)	0.3 (p12)	No abnormalities	No abnormalities
Fetus 006	M	15 + 5	*COL1A1* c.1921G>A p.(Gly641Arg) (heterozygous) (ref. seq. NM_000088.3)	0.3 (p3)	No abnormalities	No abnormalities
Fetus 005^a^	M	16 + 0	*PPIB* c.556_559del p.(Lys186Glnfs*8) (homozygous) (ref. seq. NM_000942.5)	0.7 (p40)	No abnormalities	No abnormalities
Fetus 002	F	16 + 1	*COL1A1* c.2893G>A p.(Gly965Ser) (heterozygous) (ref. seq. NM_000088.3)	<0.1 (p<1)	Except for low heart weight, no abnormalities	No abnormalities
Fetus 004	M	21 + 0	*COL1A1* c.3830A>T (p.Asp1277Val) (heterozygous) (ref. seq. NM_000088.3)	2.3 (p20)	No abnormalities	No abnormalities
Fetus 003^a^	F	22 + 1	*PPIB* c.556_559del p.(Lys186Glnfs*8) (homozygous) (ref. seq. NM_000942.5)	2.4 (p12)	No abnormalities	No abnormalities

Abbreviations: F; female; M, male; p, percentile.

a Fetus 3 and 5 are siblings. None of the other fetuses are related.

FFPE tissue was cut in 5 μm thick sections and routinely stained with hematoxylin and eosin (HE) and Elastic van Gieson (EvG) to evaluate heart microscopy. Immunohistochemical staining was performed as previously described.^15^ Immunohistochemistry was performed with primary antibodies targeting type I collagen (COL1, 1:20, Usbio, C7510-17K), collagen type I alpha 1 chain (COL1A1, 1:100, Abnova, PAB17205), and collagen type I alpha 2 chain (COL1A2, 1:100, Sigma, SAB2100463). Immunoreactivity was developed using 3,3′-diaminobenzidine (DAB, 1:50, DAKO). Light microscopy pictures were taken with a Leica DM6000B microscope (Leica microsystems). Pictures were acquired at 200× final magnification using “ViewScanner” with 600 ppi resolution, width 2592 pixels (2592 × 2076 × 24 BPP).

## Results

The 6 OI type II fetuses had a gestational age (GA) ranging from 14 weeks and 5 days to 22 weeks and 1 day, as detailed in Table 1. Segmental examination of the heart-lung block showed no anatomical abnormalities. In particular, valvular and septal defects were absent. One fetus had a small heart with a low heart weight, while 5 had a normal heart size. The fetus with low heart weight exhibited generalized growth retardation, and all organs, with the exception of the liver, had low weight according to the gestational age. The placenta of this fetus weighted 55 g (24th percentile) and displayed no abnormalities. Microscopic analysis excluded myocardial disarray and endocardial fibroelastosis. The histoarchitecture of coronary arteries, arterioles, and capillaries within the myocardium was normal.

**Figure 1.**
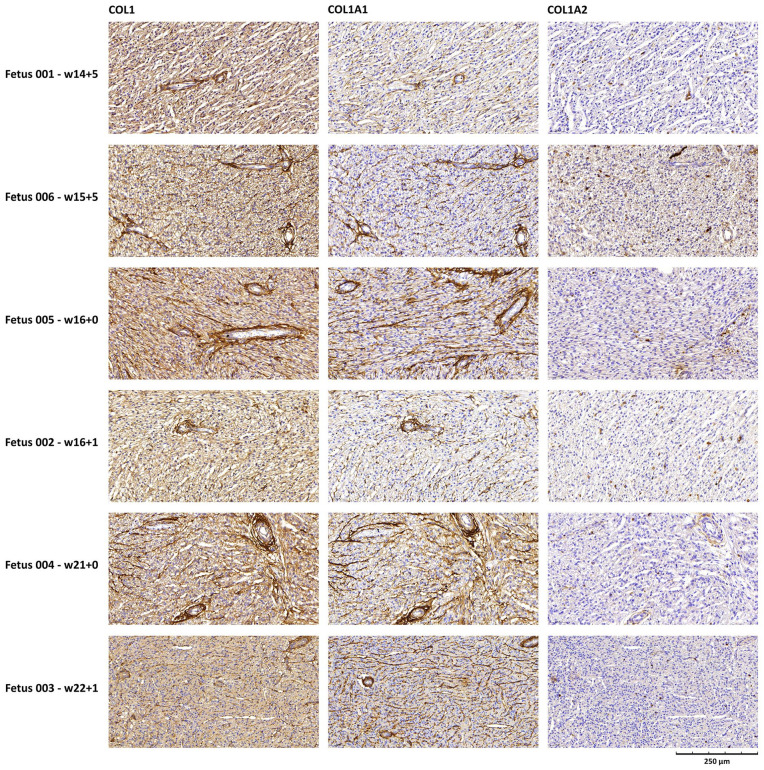
Collagen type I, COL1A1 and COL1A2 expression in the myocardial tissue of 6 OI type II fetuses investigated by immunohistochemistry. Collagen staining is shown in brown. Scale bar indicates 250 μm, and applies to all images. Gestational age is represented as weeks (w) + days.

**Figure 2.**
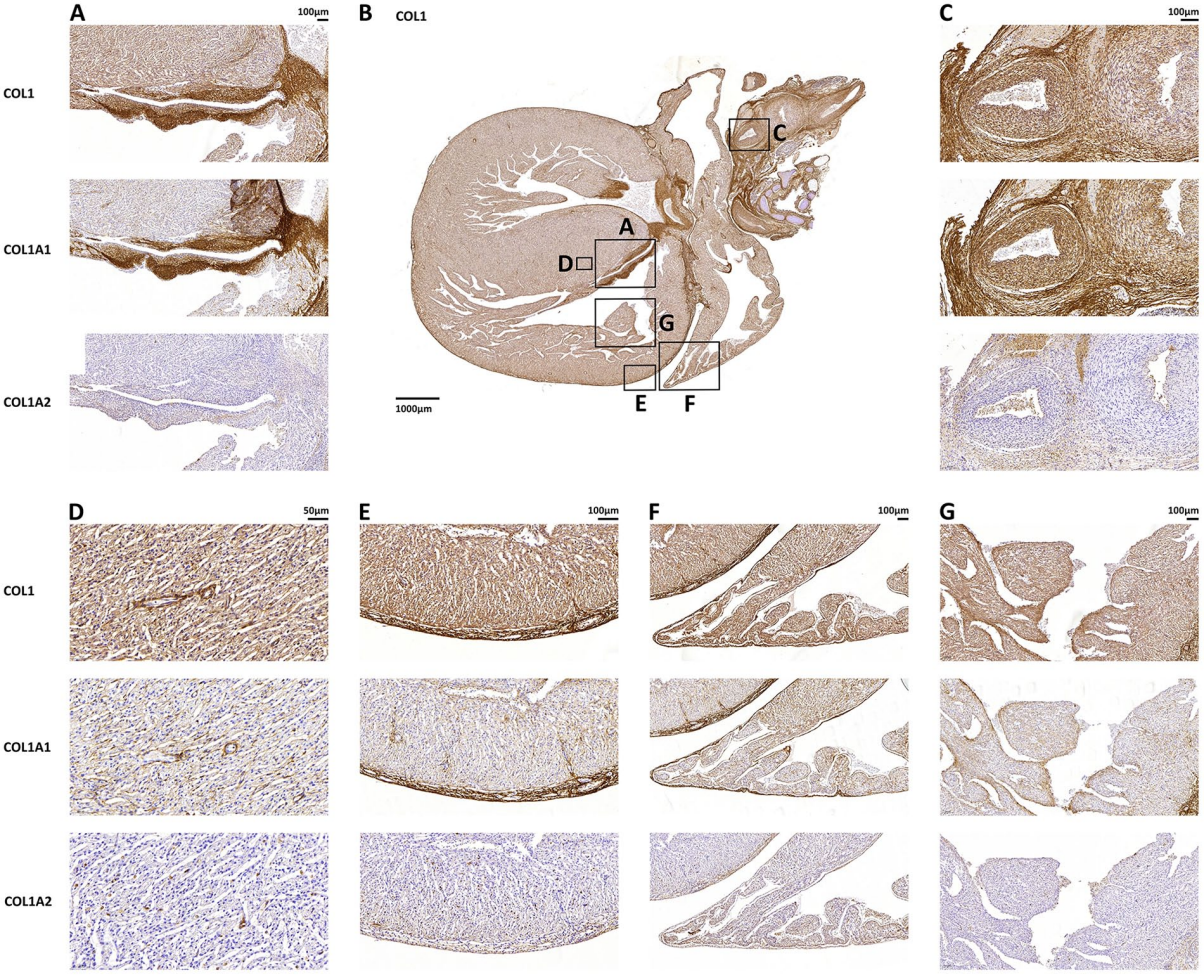
Immunohistochemical staining of collagen type I, COL1A1, and COL1A2 in the heart of OI fetus 1 (14 weeks + 5 days). Collagen staining is depicted in brown, indicating the presence of collagen type I (and its chains) in the following areas: (A) atrioventricular valve, (C) aorta, (D) myocardium, (E) epicardium, (F) atrium, and (G) endocardium. Scale bars for each anatomical location are provided. Collagen type I was found throughout the heart (B), with enrichment observed at the epicardial and endocardial surfaces, as well as in the atrioventricular valve and aortic root.

Immunohistochemistry showed that collagen type I was diffusely expressed around blood vessels, including the intramyocardial capillaries, and it was enriched at the epicardial and endocardial surfaces (Figure 1). COL1A1 expression showed a similar distribution. By contrast, COL1A2 expression was barely detected. In fetus 001, an atrioventricular valve and the root of the aorta were also included in the tissue section (Figure 2). Both collagen type I and COL1A1 were abundantly expressed at these sites, more than around the intramyocardial vessels, whereas COL1A2 expression was very faint. In the aorta, the highest expression of collagen I and COL1A1 was found in the tunicae media and adventitia.

## Discussion

The current study contributes to our understanding of cardiovascular anomalies in OI type II. To investigate whether heart anomalies such as heart failure in OI are developmental or degenerative, we conducted a retrospective analysis of the hearts from 6 affected fetuses with a confirmed genetic diagnosis of OI type II. Additionally, we investigated collagen type I and its chains’ expression in the hearts of these fetuses.

No congenital heart anomalies, including valvular or septal defects, were detected in any of the 6 OI type II fetuses, with the exception of a lower heart weight observed in 1 fetus. In addition, microscopy also excluded myocardial disarray, endocardial fibroelastosis, and other possible histoarchitectural abnormalities. As OI research has primarily concentrated on skeletal manifestations, there are only scarce reports of OI type II cases in which heart pathology has been investigated. Only 3 cases of OI type II with structural heart anomalies have previously been documented. Amongst these, 1 newborn was identified with an atrial septal defect, while 2 fetuses, at 23 and 34 weeks of gestation, displayed lower heart weights compared to control fetuses of similar gestational age.^6,7^ On the other hand, some case reports that focused on the skeletal phenotype found no heart anomalies.^9,10^ In our series, 5 out of 6 fetuses displayed normal heart weights, while 1 (fetus 002) exhibited a lower heart weight (Table 1). As the heart hypoplasia in this fetus was part of generalized growth retardation, it remains unclear whether OI is the possible cause of this, or if this should partly be attributed to the normal-sized but relatively small placenta.^16,17^ Despite the small heart size and weight, like the hearts of the other 5 fetuses, no other heart anomalies were present. This observation led us to conclude that, despite the presence of severe prenatal skeletal abnormalities in OI type II fetuses at 22 weeks of gestation, heart hypoplasia does not seem a characteristic developmental feature of this condition, but more research is needed to confirm this finding.

Qualitative defects of collagen type I also cause other types of OI, including types III and IV, and some cases of OI type I. Unlike in OI type II, the collagen type I defect does not cause lethality in patients with OI type I, III, and IV. In these OI types, both children and adults have been documented with congenital cardiovascular anomalies, such as atrial septal defects, ventricular septal defects, tetralogy of Fallot, and patent ductus arteriosus.^18-22^ However, there have been only a limited number of examined cases, and due to the absence of registry-based case-control studies addressing OI heart anomalies, it remains uncertain whether these findings are coincidental or linked to the collagen type I defect. One could speculate that, aside from coincidental cases, congenital heart anomalies may also be absent in other OI types. Nonetheless, further research is needed to establish definitive conclusions in this regard. The findings of the present case series clearly illustrate that the impact of OI on cardiac development and growth differs significantly from its effects on skeletal development. While the abnormal collagen type I in the skeleton leads to evident prenatal structural abnormalities, it can be hypothesized that collagen type I defects may compromise tissue quality in early fetal development, which may have functional consequences later in life following exposure to physiological stress. Consequently, heart complications may only become apparent postnatally in less severe OI types.

To understand the possible influence of collagen defects on cardiac development, the distribution of collagen type I in the heart was investigated in our cohort of 6 OI type II fetuses. Collagen type I was found to be highly abundant in the heart of all OI type II fetuses, which is consistent with previously published findings in healthy fetuses.^4^ Collagen type I was highly localized around capillaries (Figure 1) suggesting that it plays a role in maintaining the structural integrity of the vascular tissue. The even higher expression of collagen type I in heart valves (Figure 2), although only investigated in 1 case, also supports that collagen type I may play a crucial role in the development and maintenance of valvular structures. This is in line with a nationwide study reporting increased risk of valvular disease and heart failure in adults with OI compared to the general population.^2^ Furthermore, also consistent with the literature, collagen type I was highly expressed in the vascular wall.^23^ Next, we examined the expression of COL1A1 and COL1A2 in the heart. In our series, 4 fetuses had a pathogenic variant in the *COL1A1* gene, while 2 related fetuses had a pathogenic defect in the *PPIB* gene. As these variants lead to alterations in the composition or organization of collagen type I, rather than affecting its overall expression, no clear difference between the 6 fetuses was found in the level of expression of collagen type I, as well as the COL1A1 and COL1A2 chains. While COL1A1 was expressed abundantly, similar to collagen type I, COL1A2 expression was observed very faintly in regions where COL1A1 and collagen type I were highly expressed, showing an overall low expression of COL1A2 in the heart. To our knowledge, differences in expression between COL1A1 and COL1A2 have not been previously investigated in either healthy fetal heart tissue or OI type II heart tissue. It remains difficult to draw conclusions about the pathogenic mechanism from this small study. It is however known, that proper bonding and interrelationships of collagen strands are very important not only for structure but also for development^15^ and function of the tissue. It is assumed that seemingly small deviations at a micro level may have major consequences, therefore further research into this important topic is needed.

While our study offers new insights into the cardiovascular implications of OI type II, it does have limitations. The study was retrospective and observational, as prenatal diagnosis of OI type II typically depends on ultrasound. Advances in medical technology nowadays allow the precise prenatal diagnosis through ultrasound, which is then confirmed through genetic analysis for genetic counseling purposes. In cases where these diagnostic methods provide a clear and conclusive diagnosis, there is no longer a necessity for a post-mortem examination. This means that, increasingly, post-mortem examinations are not performed when the diagnosis of OI type II has already been established. This limits the availability of material for the characterization of the extra-skeletal pathology and disease mechanisms. While this study represents the largest case series reported to date, the small sample size constrains the generalizability of our findings. Furthermore, the absence of age-matched control samples limits our ability to draw conclusions. As the hearts of these fetuses are structurally developed, any subsequent issues are likely connected to the ongoing growth and maturation of various tissue components. However, restricting the study to this gestational stage might overlook heart pathologies that emerge later. Valvular tissue continues to remodel throughout gestation and postnatally which can potentially lead to the manifestation of abnormalities in later life,^24^ even if none were identified in these early gestation fetuses.

In conclusion, our study contributes to the understanding of the cardiovascular manifestations of OI type II by providing evidence of the absence of cardiovascular anomalies during the first 22 weeks of gestation. Therefore, cardiovascular involvement in non-lethal OI types might be degenerative in nature. However, additional studies, if possible, also including OI type II fetuses in later gestational periods, are necessary to determine whether cardiac anomalies occur at later stages of development in OI. This nuanced perspective underscores the complexity of cardiovascular involvement in OI and emphasizes the need for further research on the collagen type I-related disease mechanisms which may underlie failure of extra-skeletal organs. Such knowledge could impact the clinical follow-up and treatment of patients with OI.
